# Studies on (polytrimethylene terephthalate)/graphene oxide/f-MWCNT hybrid nanocomposites

**DOI:** 10.1186/s11671-024-03966-1

**Published:** 2024-01-30

**Authors:** Abjesh Prasad Rath, P. Santhana Gopala Krishnan, Krishnan Kanny

**Affiliations:** 1 Laboratory for Advanced Research in Polymeric Materials (LARPM), School for Advanced Research in Petrochemicals (SARP), Central Institute of Petrochemicals Engineering and Technology (CIPET), Patia, Bhubaneswar, 751024 India; 2 Advanced Polymer Design and Development Research Laboratory (APDDRL), School for Advanced Research in Petrochemicals (SARP), Central Institute of Petrochemicals Engineering and Technology (CIPET), Devanahalli, Bengaluru, 562149 India; 3 Composites Research Group, Department of Mechanical Engineering, Durban University of Technology, Durban, 4000 South Africa; 4 Faculty of Engineering and the Built Environment, Durban University of Technology, P O Box 1334, Durban, 4000 South Africa

**Keywords:** Multiwalled carbon nanotubes, Graphene oxide, Thermal conductivity, Mechanical properties

## Abstract

Natural resource-driven approaches to bioengineering plastics are being developed to compete in the automobiles, power, and other sectors. Polytrimethylene terephthalate (PTT) is a particular of them, and it was chosen for the current investigation to build an advanced nanocomposite material. Using a twin-screw micro compounder, injection moulded PTT/Graphene-Oxide (GO)/Carboxyl functionalized Multiwall Carbon nanotube (f-MWCNT) hybrid nanocomposites were prepared. The impact of GO and f-MWCNT reinforcement on the composite’s thermal and mechanical characteristics of hybrid nanocomposites was examined. GO was synthesized from the graphite powder by modified Hummer’s method and MWCNTs were functionalized using the concentrated sulfuric acid (H_2_SO_4_) and nitric acid (HNO_3_) with a volume ratio of 3:1 in an ultrasonic bath at room temperature. In all formulations, the investigation was done at a constant filler amount of 2 wt%. To understand the chemical interaction between PTT and nanofiller, Raman spectroscopy was used and to examine the state of dispersion, scanning electron microscopy (SEM) was systematically analysed. In comparison to pristine PTT, the water absorption, tensile strength, flexural strength and impact strength of hybrid nanocomposites were improved marginally. It was also observed that GO has more prominent in increasing the mechanical properties of the hybrid and f-MWCNT in thermal properties. The 3-D geometrical bridge between GO (2-D) and f-MWCNT (1-D) made the hybrid more dispersible and effective for different applications.

## Introduction

PTT is a widely used thermoplastic commercial polymer having low melting temperature, and low water absorption properties with respect to Polyethylene terephthalate (PET) [1]. PTT is developed by the polycondensation reaction of 1,3-propanediol and terephthalic acid [2, 3]. But due to some limitations like fracture-brittleness, low toughness confined its applications in several fields. So, for increasing the mechanical strength of PTT, GO is used as a nanofiller for hybrid nanocomposite. Enhanced productibility and processability of GO, encourage us to use GO in nanocomposite materials. The ability to functionalize GO with different functional groups and also have free radicals, acidic properties & high catalytic activity, makes it usable in nanocomposite preparation [4]. Again, the incorporation of MWCNTs into polymer matrix improves their physical, mechanical and thermal properties for which polymer/carbon nanocomposites have been noticed by scientists and industry. But due to strong Van der Waal’s force between MWCNTs gives rise to exceedingly large aggregation which leads to non-uniform dispersion of MWCNTs and also a hindrance to producing high-quality nanocomposite [5]. The dispersion quality of MWCNTs can be improved through functionalization in the nanocomposite and also a better property can be obtained with smaller loading of MWCNTs [6]. As GO has a 2-D geometrical structure and f-MWCNT has a 1-D structure, when they disperse together with the polymer matrix, it forms a 3-D chain-like network that gives a better reinforcement with the polymer and forms a composite of highly improved mechanical and thermal properties [7]. A considerable amount of research has been conducted to examine PTT’s thermal and crystallization behaviours, fiber characteristics, and crystal structure [8–10]. Various composite systems, including PTT/GF, PTT/clay nanocomposites, PTT/carbon nanotubes, and PTT/BaSO_4_, were researched and reported to enhance their characteristics [11–14]. PTT is a thermoplastic polyester that exhibits good thermal properties and processability. Generally when combined with highly thermal conductive additives like graphene and MWCNT, the resulting nanocomposite material have enhanced thermal conductivity. In our present study, PTT reinforced with GO and f-MWCNT, also have enhanced thermal conductivity and hydrophilicity which can be advantageous in applications where there is a requirement of these enhanced properties.

## Experimental

### Materials

PTT was supplied by Futura Polyesters Ltd., Chennai, India. GO and f-MWCNT were prepared and characterized as reported elsewhere [15].

### Fabrication of the PTT/GO/f-MWCNT hybrid nanocomposites

Nanocomposites were prepared using a Haake mini jet pro twin-screw extruder coupled with an injection moulding system from thermo-fisher, USA. Prior to composite formation, the granules of neat PTT were dried in a hot air oven at 80 °C for 48 h. Nanocomposites of PTT/GO/f-MWCNT were prepared with 0, 0.5, 1.0, 1.5 and 2 wt% nanofiller. About 30gm of nanocomposite mixture (PTT and nanofiller) was compounded at 50 rpm and 250 °C for 5 min and then the nanocomposite mixture was transferred to a preheated cylinder of temperature same as extruder. Then the standard specimens were prepared by an injection moulding system by putting mould temperature 25 °C and pressure 5 bars for 20 s before demoulding. Prior to characterization, the specimens were conditioned for 24 h at a relative humidity 50 ± 5%.

### Testing and characterization

On a Horiba Jobin Yvon T64000 that has a CCD symphony detector and an Olympus microscope stage, Raman spectra were captured. Spectra of PTT and its hybrid nanocomposites were acquired using the spectrometer in the triple subtractive mode over the frequency range of 2900–500 cm^−1^. A Coherent INNOVA-400 argon ion laser’s 514.5 nm line was employed for excitation. A 15 mW of laser energy was directed at the sample to evaluate the Raman spectra of pure PTT and its hybrid nanocomposites. Fourier transform infrared spectrometry (FTIR; Intertek 58,595; EVO MA 15; Cari Zeiss SMT, Germany) was used to investigate the grafting reactions of GO and f-MWCNT onto PTT and to verify ester bond formation between the f-MWCNT, GO and the PTT matrix. FESEM images were collected by an EVO MA 15, Cari Zeiss SMT (Germany) scanning electron microscope. The samples of FESEM imaging were coated with a thin layer of gold film to avoid charging.

Percentage of water absorption of PTT and its nanocomposites was calculated by difference between final weight of specimen after removing from water (${\text{w}}_{{\text{f}}}$) and initial weight of the specimen before putting in water (${\text{w}}_{{\text{i }}}$) and then divided by initial weight. So before putting the specimens in the water for 24 h, the samples were just dried by putting them in a hot air oven at 80 °C for 24 h and after that cooling in a desiccator to calculate the exact initial weight. After 24 h putting in water, the specimens were wiped by a lint free garment to dry them. So, here is the equation for calculation of % of water absorption.

1 $${\text{w}} = \frac{{\left( {{\text{w}}_{{\text{f}}} - {\text{w}}_{{\text{i }}} } \right)}}{{{\text{w}}_{{\text{i}}} }} \times 100$$

PTT and its nanocomposites were tested for intrinsic viscosity at 25 °C (standard ASTM 2857) using an Ubbelohde viscometer and capillary I_c_ (K = 0.03294) with polymer solutions of 0.5 g/dL in phenol/trichloroethylene (60/40 by weight). The Mark–Houwink equation relates M_v_ and [η] to intrinsic viscosity (IV), often known as the limiting viscosity number [η].

2 $$\left[ \eta \right] = {\text{K}}\cdot{\text{M}}^{\alpha }_{{\text{v}}}$$

where K and α are constants that depend on the temperature and solvent, respectively. Using the following constants, the viscosity average molecular weight ($\overline{\text{M}}_{\text{v}}$) of PTT and its nanocomposites was calculated using K = 5.36 × 10^–4^ dl/g and α = 0.69 as reported in the literature [16].

A Krus G10 device was used to calculate the contact angle of PTT and its nanocomposites. Samples were inspected on six different sites of the plane flat specimen to get an average reported value of contact angle of each sample.

Differential scanning calorimetric (DSC) analysis of PTT and its nanocomposites were performed using the Hitachi DSC 7020 instrument to examine their melting and crystallization behaviour in a nitrogen atmosphere. The samples were heated from room temperature to 250 °C at a rate of 10 °C/min. The area of the DSC endotherm and exotherm, respectively, was used to compute the heats of fusion (ΔH_m_) and crystallisation (ΔH_c_) respectively. Equation (3) refers to the ratio of the integrated heat of fusion ΔH_m_ value of the sample over the heat of fusion of totally crystalline PTT, i.e., ΔH°_m_ = 145.6 J/g [17], was used to measure the crystallinity % (X_c_), or the weight fraction crystallinity.

3 $${\text{X}}_{{\text{c}}} \left[ \% \right] = \frac{{\Delta H_{m} }}{{\Delta H^\circ_{m } }}$$

The heat deflection temperature (HDT) was reported as the temperature at which a deflection of 0.25 mm occurred. It was measured under 1.8 MPa with reference to ASTM D648. The thermal conductivity measurements were carried out at room temperature using the hot disk thermal constant analyzer (TA Instruments, USA) using ASTM C518 test method.

Tensile and Flexural analysis of the hybrid nanocomposites were carried out by adopting ASTM D638 and ASTM D790 respectively, using a DAK system UTB9103 universal testing machine (UTM). The type 5 bone shaped specimens, with gauge lengths of 7.62 mm, were prepared by a micro-injection machine. The tensile and flexural specimens were stressed at a rate of 1 mm/min and 1.4 mm/min respectively. The impact analysis was performed through a notched Izod impact test (where the notch depth was 4.0 mm) employing TMI 43–02 Monitor Impact Tester concerning ASTM D256. Using the dynamic mechanical analyzer (DMA) Discovery DMA580 from TA Instruments, the thermo-mechanical characteristics of PTT and its hybrid nanocomposites were assessed using rectangular composite bars with dimensions of 50 mm × 12 mm × 3.25 mm. Using dual cantilever mode, a heating rate of 5 °C/min, a 15-micron amplitude, and a 1 Hz frequency, the storage modulus, loss modulus and tan δ were measured.

## Result and discussions

PTT hybrid nanocomposites with various f-MWCNT and GO weight percentages were coded. Table 1 lists the code for various nanocomposites. The total amount of nanofiller was kept constant at 2 wt%. Here, GO is referred to as G and f-MWCNT as M.

**Table 1:** Physical properties of PTT nanocomposites

Composite	GO (wt%)	f-WCNT (wt%)	% water absorption
PTT	0	0	0.0982
M2	0	2	0.1013
G0.5M1.5	0.5	1.5	0.1027
G1M1	1	1	0.1079
G1.5M0.5	1.5	0.5	0.1216
G2	2	0	0.1252

### Physical properties

The measure of water absorption is the amount of water consumed by the hybrid nanocomposites over a 24-h period at room temperature, as determined by standards. The greater the water absorption, the greater the water penetration into the nanocomposites, and the worse the moisture barrier qualities of the final product [18]. The inclusion of GO and f-MWCNT combined boosted water absorption in the current investigation. The water absorption percentage for neat PTT at room temperature was 0.0982%, whereas the water absorption value of nanocomposites rose with the introduction of nanofiller content, indicating that the permeability of water in nanocomposites enhanced.

However, water absorption experiments supported the promising behaviour of GO in enhancing material permeability, which is 0.1252% for G2 as a 27.5% increase. Because GO is hydrophilic and is composed of oxygen groups, that improve PTT nanocomposite water absorption. The high-water absorption capacity may be useful in certain applications.

### Raman spectroscopy of PTT its nanocomposites

**Fig. 1:**
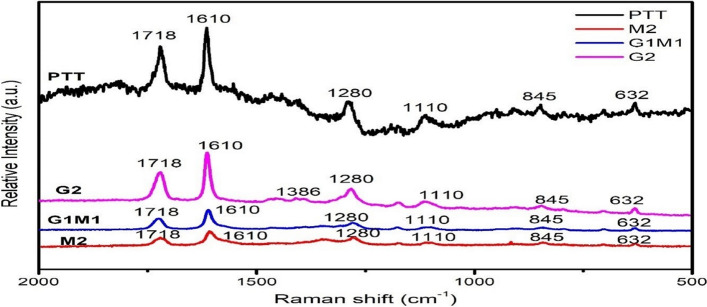
Raman spectroscopy of **a** PTT **b** G1M1 **c** M2 **d** G2

Figure 1 depicts the Raman spectra of nanocomposite specimens. The Raman D and G bands at 1386 and 1610 cm^−1^, respectively, demonstrate the occurrence of the *sp*^2^-hybridized GO and f-MWCNT frameworks [19]. Due to band overlaps with bands attributed to the polymer matrix, the D band, which is located at 1386 cm^−1^, cannot be recognised clearly. However, the low intensity of the D band in contrast to the G band suggests that f-MWCNTs and GOs are of a high purity. When compared to G2 and G1M1, the relative intensity of the G band is lower in the M2 nanocomposite. The intensity of the 2D band reported for M2 does not differ considerably from that shown for G2 at 1280 cm^−1^.

The 2D band resulting from a double resonance process is no longer as evident for G2, as expected after significant functionalization and disruption of the p-conjugated network [20]. It indicates the inclusion of f-MWCNTs and GO within the PTT nanocomposite results in greater functionalization, maybe as an effect of free radical assault on the basal planes of the GO and the sidewalls of the MWCNTs, leading to further extensive functionalization. Like that the peaks at 845 cm^−1^ and 632 cm^−1^ shows the C–C stretching (ring breathing) and CCC in plane bending (ring) respectively [21]. Also peak at 1718 cm^−1^, corresponding to the carbonyl stretching of the hybrid nanocomposites.

### FTIR analysis of PTT and its nanocomposites

**Fig. 2:**
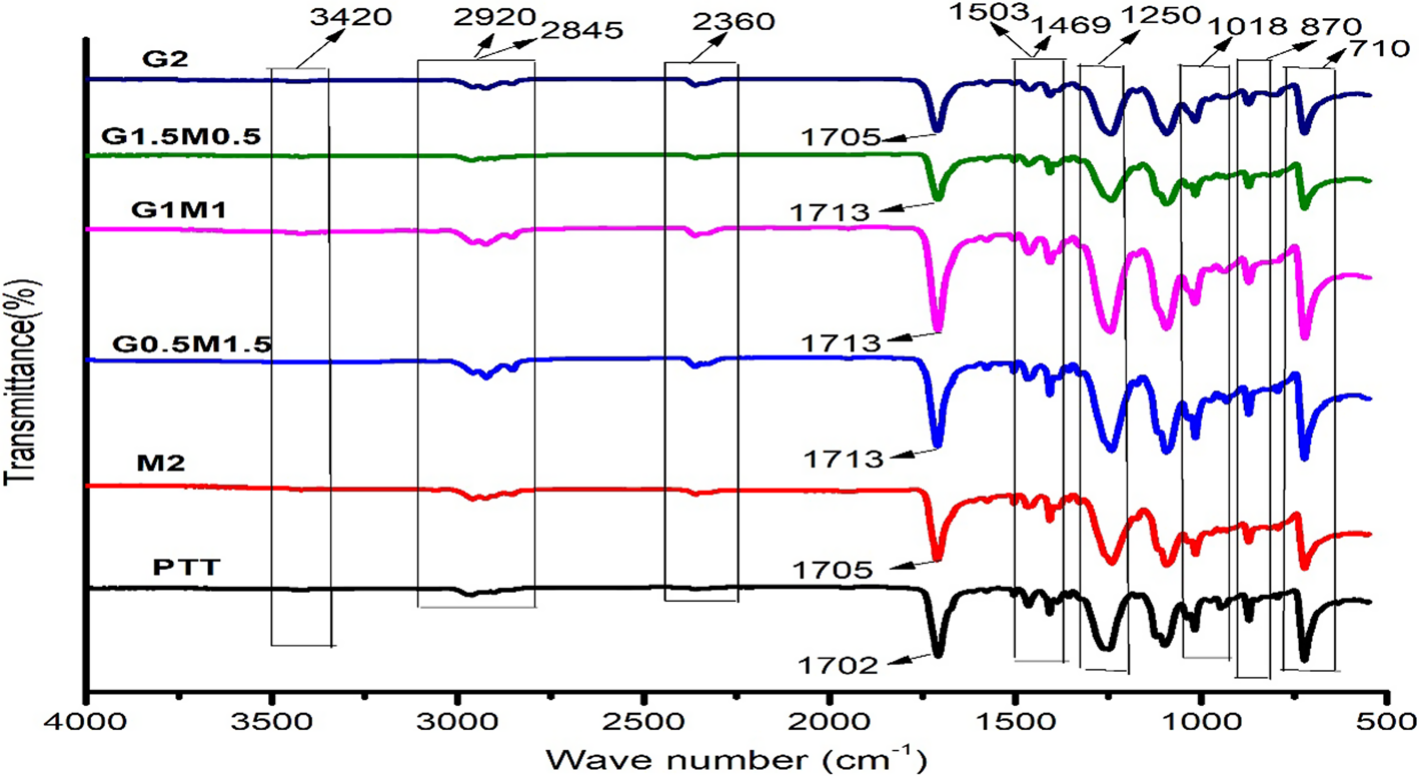
FTIR spectra of PTT and its nanocomposites

FTIR studies were taken to investigate the structure of the PTT and its hybrid nanocomposites. Figure 2 depicts the FTIR spectra of clean PTT, M2, G0.5M1.5, G1M1, G1.5M0.5, and G2. The stretching vibration of the C=O group in the ester bond of PTT was illustrated by the peak at 1702 cm^−1^ and the peak at 710 cm^−1^ was caused by three methylene(–CH_2_) groups [22]. Peak nearer to 1250 cm^−1^ was assigned to the stretching vibration of the C­O in the ester group. The bands at 2845 and 2920 cm^−1^ were assigned to the symmetric and asymmetric stretching vibration of the –CH_2_– group. The vibrations at 1503 cm^−1^ and 1469 cm^−1^ belong to phenyl group C=C stretching vibrations, while the oscillations at 1018 and 870 cm^−1^ relate to benzene ring C–H stretching fluctuations. [23]. A shift in the carbonyl stretching frequency from 1702 to 1705 cm^−1^ in case of M2 and G2 and to 1713 cm^−1^ in case of G0.5M1.5, G1M1 and G1.5M0.5 is an indication of changes in hydrogen bonding interactions. Strengthening or weakening of hydrogen bonding caused shifts in the absorption band as shown in Fig. 2. Similar observation was reported by Manju et al. for justifying the reaction occurred between Poly(lactic acid)-Sepiolite nanocomposites [24].

The presence of OH and/or COOH functional groups indicates the existence of an extremely faint band at 3420 cm^−1^, showing the prevalence of GO and f-MWCNT within structure [25]. Also a band at 2360 cm^−1^ may be due to the axial symmetric deformation of CO_2_ group. A band at 712 cm^−1^ was due to interaction of polar ester groups and benzene rings. As there were no notable changes in the position of peaks of pristine PTT after blending with GO and f-MWCNT which proved that only physical interaction happened between matrix and nanofillers.

### Intrinsic viscosity

Nanocomposites have higher intrinsic viscosities than pristine PTT, according to measurements of these materials viscosity as shown in Table 2. Additionally, the increase in viscosity is significantly influenced by GO content rather than f-MWCNTs content. The fact that nanocomposites have higher intrinsic viscosity than pristine PTT suggests that network structures made of f-MWCNT-f-MWCNT, f-MWCNT-GO, and GO polymer connections have developed. In the PTT/f-MWCNT nanocomposites, the COOH functional groups on the surface of MWCNTs can engage with the ester groups of PTT molecules by hydrogen bonding.

**Table 2:** Calculation of average molecular weights by the Mark–Houwink method

Sample code	Intrinsic viscosity (dL/g)	$\overline{\text{M}}_{\text{v}}$ (g/mol)
PTT	0.95	51,058
M2	0.96	51,839
G0.5M1.5	0.98	53,412
G1M1	1.03	57,400
G1.5M0.5	1.13	65,650
G2	1.18	69,906

In Poly(ethylene 2,6-naphthalate) (PEN)/Carbon nanotube (CNT) composites, the existence of hydrogen bonds between the functional groups of CNT and polyester molecules has been confirmed. According to tests of the intrinsic viscosity number, the average viscosity molecular weight ($\overline{\text{M}}_{\text{v}}$) of neat PTT and its nanocomposites ranges from 51,000 to 70,000 g/mol. Viscosity Average molecular weight of nanocomposites increased in comparison to neat PTT due to sufficient chain entanglement and low molecular mobility after reinforcement of GO and f-MWCNT in the polymer matrix.

### FE-SEM analysis

Morphology of PTT nanocomposites containing GO and f-MWCNT was investigated by FE-SEM analysis. Controlling the extent of dispersion of MWCNTs in a polymer matrix can be challenging due to the high intermolecular interactions that exist between carbon nanotubes, which are involved in the production of nanotube bundles. These bundles are hard to exfoliate because the nanotubes can be hundreds to thousands of nanometres long, disrupting the even distribution of nanotubes in a polymer matrix. [26].

**Fig. 3:**
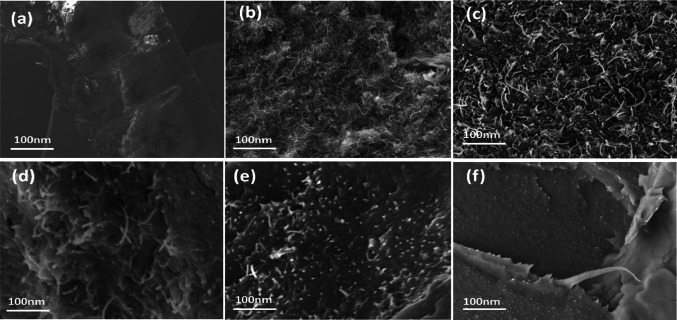
FESEM images of **a** Neat PTT **b** M2 **c** G0.5M1.5 **d** G1M1 **e** G1.5M0.5 **f** G2

As illustrated in Fig. 3a, the PTT polymer has a smooth fracture surface. Figure 3b depicts FE-SEM micrographs of the shattered surface of PTT nanocomposites including f-MWCNT. As previously stated, CNTs frequently bundle together due to Van der Waal’s interaction between individual nanotubes with high aspect ratios and large surface areas, resulting in certain agglomerations that limit efficient load transfer to polymer matrix [27]. It was found from Fig. 3f that the graphene flakes were evenly dispersed throughout the PTT matrix, but that some small aggregates did form at higher GO concentrations. This resulted from both the GO’s extremely high specific area and the robust particle–matrix interactions that exist. As shown in Fig. 3c, d and e the GO with f-MWCNT showed some bridge moieties morphology associated with the formation of hierarchical nanostructure which could assist in MWCNTs intercalation. Compared with those of M2 and G2, hybrid nanocomposites G0.5M1.5, G1M1 and G1.5M0.5 showed good surface roughness.

### Mechanical properties

**Fig. 4:**
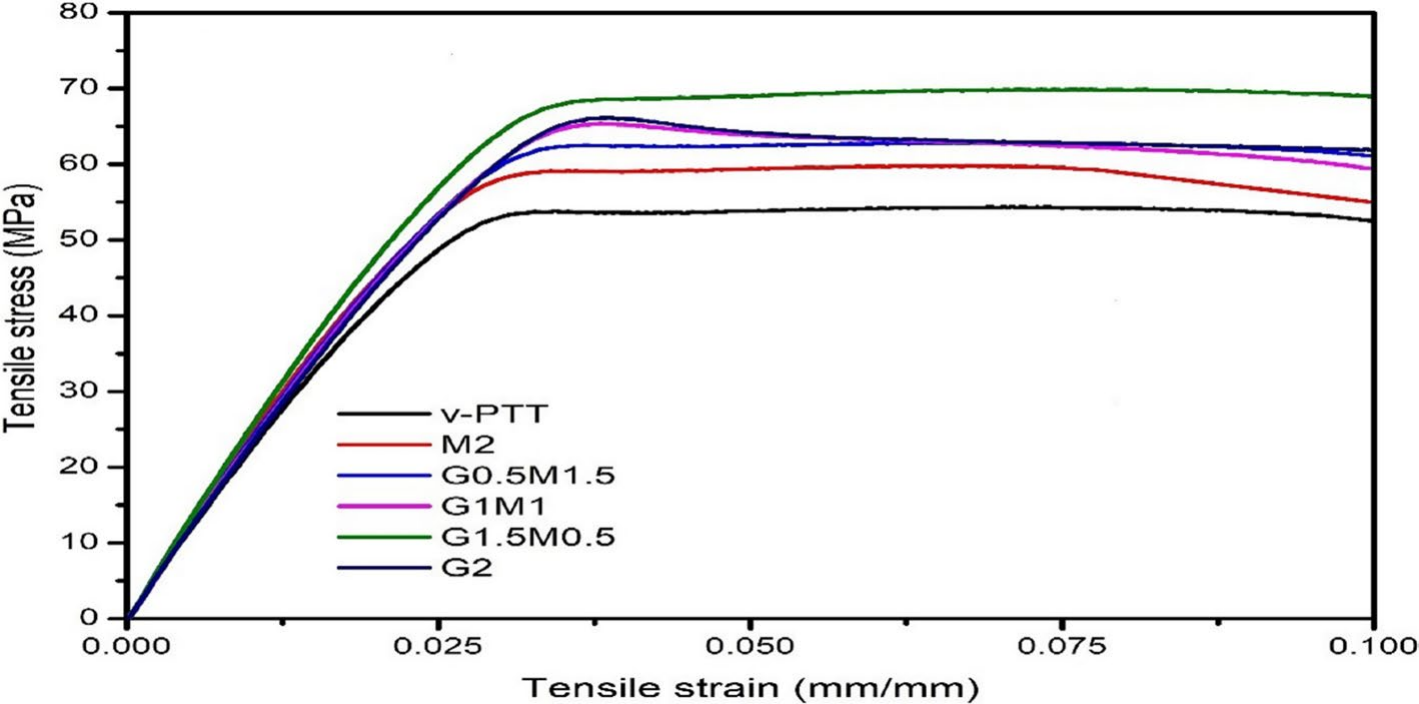
Stress versus Strain curve for virgin PTT and its nanocomposites

The typical stress–strain curves for PTT and its nanocomposites are shown in Fig. 4. In contrast to PTT/GO/f-MWCNT nanocomposites, neat PTT manifests the lowest stress value and exhibits a large amount of deformation with a yield strength region. Table 3 displays the tensile characteristics of PTT and its nanocomposites with varying nanofiller loadings. Because of the strong interphase contact between the PTT and the GO and f-MWCNT, the tensile strength of hybrid nanocomposites enhanced significantly. The interphase in PTT nanocomposites was affected by Van der Waal’s forces between MWCNTs and PTT matrix, but additional effects could arise from hydrogen bond interactions between COOH functional groups on the surface of MWCNTs and ester groups of PTT molecules, as well as interactions between the oxide groups of GO and benzene rings of the terephthalate moiety.

**Table 3:** Tensile data of PTT and its nanocomposites

Sample code	Tensile strength (MPa)	Tensile modulus (GPa)	Elongation at break (%)
PTT	50 ± 1	2.2 ± 0.1	4.3 ± 0.3
M2	54 ± 5	2.3 ± 0.2	4.2 ± 0.7
G0.5M1.5	56 ± 3	2.6 ± 0.4	4.2 ± 0. 6
G1M1	59 ± 2	2.8 ± 0.3	4.1 ± 0.9
G1.5M0.5	65 ± 2	3.2 ± 0.5	3.8 ± 0.1
G2	61 ± 4	2.9 ± 0.1	3.6 ± 0.5

The tensile strength of G1.5M0.5 was enhanced to 65 MPa, which is approximately 30% greater than the tensile strength of neat PTT (~50 MPa). A similar observation was shown by Braga et al. that PTT matrix was modified with a compatibilizer agent based on maleic anhydride grafted PTT (PTT-*g*-MA) and functionalized CNT, showed an increase in tensile strength of about 18% as compared to neat PTT [28].

Like that the modulus of the PTT/GO/f-MWCNT increased with increasing loading of GO except G2 and decreasing loading of f-MWCNT and maximum modulus was found to be 3.2 GPa in case of G1.5M0.5. Also, it was seen from Table 3 that addition of f-MWCNT and GO had not affected elongation at break and remained almost same. No change in % elongation at break was seen in the nanocomposites. Reinforced nanocomposites frequently exhibit this quality. The restriction in the polymer chain’s mobility brought on by the stiff nanofiller particles is attributed for the no effect in elongation at break [29].

Flexural analysis of PTT/GO/f-MWCNT hybrid nanocomposites was executed with different GO and f-MWCNTs loading and results of flexural strength and flexural modulus are shown in Table 4. The calculated values show that the flexural strength and flexural modulus increased with increasing GO loadings and with decreasing f-MWCNT loadings. Flexural strength increased from 72.2 to 89.7 MPa which is about 24% higher than neat PTT and Flexural modulus increased from 2.3 to 2.7 GPa for the G1.5M0.5 reinforcement which is about 17% higher than neat PTT. Observed flexural strength and flexural modulus with hybridization of GO and f-MWCNT indicate a good 3-D interaction between nanofillers and the PTT matrix which was effective in stress transfer from matrix to filler.

**Table 4:** Flexural and impact data of PTT and its nanocomposites

Sample code	Flexural strength (MPa)	Flexural modulus (GPa)	Impact strength (J/m)
PTT	72.2 ± 1	2.3 ± 0.3	6.0 ± 1
M2	74.1 ± 3	2.4 ± 0.5	8.6 ± 0.2
G0.5M1.5	76.5 ± 5	2.5 ± 0.1	9.2 ± 0.8
G1M1	78.9 ± 2	2.5 ± 0.2	10 ± 0.6
G1.5M0.5	89.7 ± 1	2.7 ± 0.4	11.5 ± 0.5
G2	81.3 ± 3	2.6 ± 0.3	9.8 ± 0.4

Notch Impact strength of PTT/GO/f-MWCNT hybrid nanocomposites with different nanofiller loadings are shown in the Table 4. The Impact strength of the hybrid nanocomposite materials indicates the resistance against crack growth. Impact strength increased upon addition of single or hybrid nanofiller. The percentage of nanofiller and the composition of nanocomposite has effect on the impact strength of nanocomposites.

### Dynamic mechanical analysis (DMA)

**Fig. 5:**
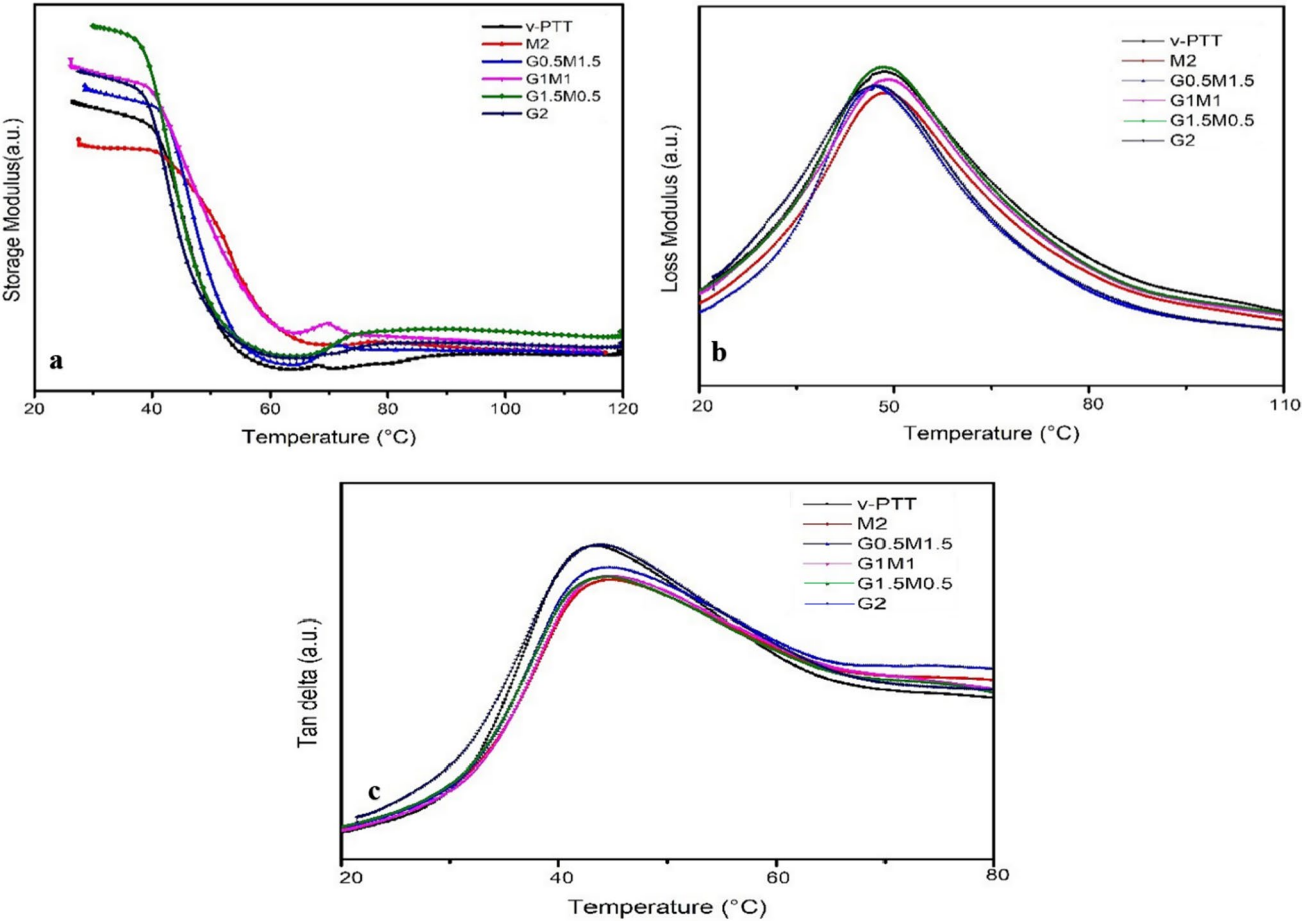
Evolution of **a** storage modulus, **b** loss modulus and **c** tan δ with temperature for PTT and its nanocomposites

Figure 5a depicts the storage modulus versus temperature graph. When contrasted to pristine PTT, these data reveal that the storage modulus of PTT improved with the addition of GO and f-MWCNT, with the largest boost found with G1.5M0.5. This conclusion is compatible with the tensile and flexural modulus, as crystallization has the greatest effect on composite modulus. The modulus decreases sharply as the temperature rises as a result of the reorganization of mobile molecules [12]. It was also found that the peak value of tan δ of glass transition region increased upon addition of GO and f-MWCNT as shown in Fig. 5c.

The storage modulus of PTT at room temperature was found to be 1217 MPa, which increased to 1670 MPa (for G1.5M0.5), which is 37% greater than the neat PTT as reported in Table 5. As explained in the preceding sections, the same patterns were detected in the tensile and flexural modulus of PTT/GO/f-MWCNT nanocomposites. The findings observed for PTT/carbon fiber composites were similar to the literature published by Singaravelu et al. [30]. As a result, the inclusion of GO and f-MWCNT to the polymer matrix increased their stiffness, resulting a hike in the modulus of the nanocomposites. The damping capabilities of carbon filler-reinforced PTT are mostly affected by its stiffness. The reduced peak values of loss modulus curve as shown in Fig. 5b indicates that the incorporation of f-MWCNTs and GOs into PTT matrix decreased the molecular mobility, which causes a reduction in their damping ability.

**Table 5:** Viscoelastic characteristics of PTT and its nanocomposites

Sample code	Storage modulus (MPa)	Loss modulus (MPa)	Glass transition temp. (°C)
PTT	1217	164.8	43.0
M2	1368	130.1	48.5
G0.5M1.5	1449	130.8	45.1
G1M1	1548	143.5	44.2
G1.5M0.5	1670	185.6	48.9
G2	1537	138.7	46.3

### Crystallization and melting behaviour of nanocomposites

**Fig. 6:**
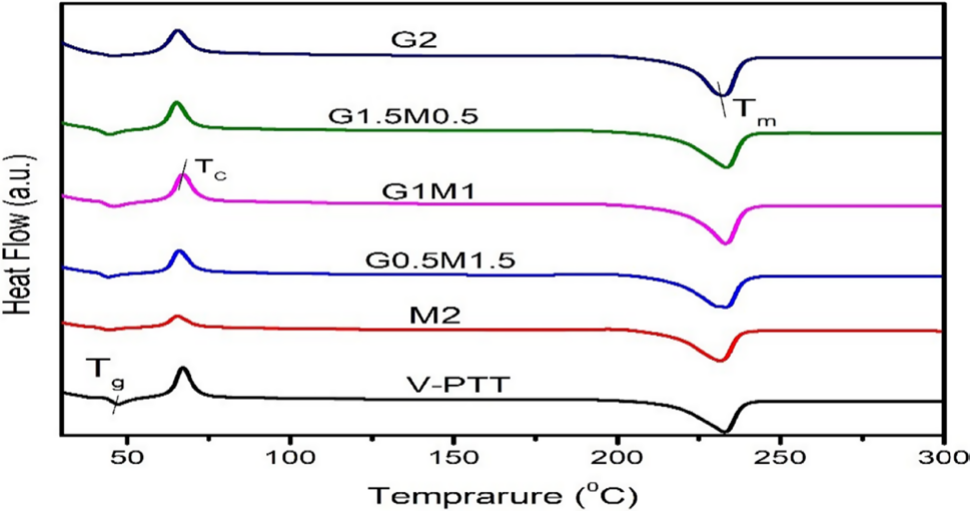
Melting and crystallinity curve of PTT and its nanocomposites

Differential scanning calorimetric thermograms of PTT/GO/f-MWCNT hybrid nanocomposites with different nanofiller loading are shown in Fig. 6. As discussed in above the increase in viscosity molecular weight which ultimately caused increase in value of glass transition temperature (T_g_) from neat PTT of 44.5–46 °C in case of G1M1 as shown in Table 6. The enhancement in melting temperature (T_m_) from 231.7 °C for neat PTT to 235 °C for G1.5M0.5, proved that nanofillers reinforced in the polymer could prevent the conduction of heat to crystallites but the heat flow was sufficient to melt down the crystallites. DSC curve shows that the crystallization temperature of PTT increased massively due to the addition of GO and f-MWCNT, which confirms the nucleating effect of nanofillers on PTT. Nanofillers usually affect the crystallinity of semi-crystalline polymers and these are distributed in the nanocomposites assisted by nucleation and growth of crystallites [31]. In this work, there was a very small effect of nanoparticles on the physical transitions of PTT. There may be a problem of establishing active centres for the growth of crystallites due to the sizes of individual GO and f-MWCNT which was below the critical nucleation agents. The alignment of mobile polymer chains leads to polymer crystallisation.

**Table 6:** The data for melting and crystallinity of the prepared samples

Sample Code	T_g_ (°C)	T_c_ (°C)	ΔH_c_ (J/g)	T_m_ (°C)	ΔH_m_ (J/g)	X_C_ (%)
PTT	44.5	65.3	53.7	231.7	73.1	50.2
M2	47.3	66.9	50.3	232.9	72.5	49.8
G0.5M1.5	44.5	66.7	53.5	233.4	73.2	50.3
G1M1	46.0	66.8	54.0	233.2	74.1	50.9
G1.5M0.5	45.6	67.7	54.4	235.0	75.4	51.8
G2	45.8	65.4	52.8	232.5	72.8	50.0

The introduction of nanofiller to polymer composites modifies the thermal properties of the materials because of the formation of interfacial interactions between the surfaces of the nanofiller and the polymer. The links, which can be established via chemical, physical, or a combination of the two, restrict the flexibility of the polymer chains. The rise in the degree of supercooling in this case is due to the introduction of short and thin f-MWCNTs and GO into the PTT matrix. These may be brought on by either their small aspect ratio or the potent interactions they have with PTT chains. About 0–2 °C less of the crystallisation peak temperature (T_c_) is reduced. Usually, lower supercooling levels correspond to faster crystallisation and nucleation rates.

No discernible change in the % crystallinity was seen in the prepared nanocomposites, and the values for the hybrid nanocomposite with the highest concentration of f-MWCNTs + GO and nanocomposites with the same concentration of both f-MWCNT and GO. This was probable because the diameters of individual GO nanoplatelets and carbon nanotubes were smaller than the necessary nucleation agents, which may have prevented them from acting as active crystallite growth centres.

### Heat deflection temperature (HDT)

The influence of f-MWCNT and GO on HDT values was investigated, and the results are displayed in Table 7. The HDT of nanocomposites has been found to be greater than immaculate PTT, reaching a maximum of 49.8 °C for G1M1, which is 8.1 °C (20%) higher than the clean PTT (41.7 °C). According to Liu et al., the insertion of glass fiber into PTT enhanced the HDT by causing an increase in modulus [32]. In these nanocomposites also, both HDT and modulus were higher than the neat PTT. As both f-MWCNT and GO are known for their reinforcing properties, the addition of these nanofillers improved the overall structural integrity and resistance to deformation of the PTT matrix which was also reported in DSC data of nanocomposites.

**Table 7:** HDT and Thermal Conductivity of clean PTT and its nanocomposites

Sample code	HDT (°C)	Thermal conductivity (W/mK)
PTT	41.7 ± 2	0.212
M2	42.0 ± 3	0.240
G0.5M1.5	43.0 ± 5	0.223
G1M1	49.8 ± 1	0.215
G1.5M0.5	43.8 ± 4	0.218
G2	42.5 ± 5	0.214

HDT of G1M1 seems to be an aberration from other samples because at equal wt%, the two nanofillers might be interacting with each other and the PTT matrix in unique ways, leading to improved dispersion and interfacial adhesion. This could enhance heat transfer within the material, resulting in higher HDT values.

#### Thermal conductivity

Table 7 shows the thermal conductivity of Neat PTT and PTT-based hybrid nanocomposites filled with f-MWCNT and GO, generated by melt compounding. It was discovered that the inclusion of both carbon nanofillers increased the heat conductivity of the polymer matrix. Furthermore, when compared to GO, the addition of f-MWCNT resulted in much larger enhancement as the f-MWCNT nanofiller percentage increased. The existence and properties of a crystalline structure of PTT, which is believed to operate as a medium of heat transfer in both the polymer phase at the interface between CNT and polymer, are responsible for the substantial rise in the thermal conductivity of M2 [33].

## Conclusions

Fabrication of PTT/GO/f-MWCNT hybrid nanocomposites with different nanofiller loadings was successfully performed through a twin-screw extruder. The Raman spectroscopy identified GO and f-MWCNT with relevant graphite and MWCNT. FTIR revealed that there have been only hydrogen bonding interactions between PTT matrix and GO, f-MWCNT fillers, as peak changes were observed in carbonyl peak. GO and f-MWCNT reinforcement increased the crystallization temperature of PTT which indicating the nucleation effect of carbon nanofillers on the PTT matrix. Mechanical properties of PTT/GO/f-MWCNT hybrid nanocomposites were enhanced with increasing GO content. Flexural strength and flexural modulus were improved by 21% and 13% respectively for G1.5M0.5. A 38% improvement of the impact strength was observed for PTT/GO/f-MWCNT hybrid nanocomposites with G1.5M0.5. From this, it is concluded that PTT/GO/f-MWCNT hybrid nanocomposites have excellent potential in applications where there is requirement of enhanced hydrophilicity and thermal conductivity.

## Data Availability

The datasets generated during and/or analysed during the current study are available from the corresponding author on reasonable request.

## Code availability (software application or custom code)

Yes.
